# Big data and ICT solutions in the European Union and in China: A comparative analysis of policies in personalized medicine

**DOI:** 10.1177/20552076221129060

**Published:** 2022-10-26

**Authors:** Francesco Andrea Causio, Ilda Hoxhaj, Flavia Beccia, Marzia Di Marcantonio, Timo Strohäker, Chiara Cadeddu, Walter Ricciardi, Stefania Boccia

**Affiliations:** 1 Section of Hygiene, University Department of Life Sciences and Public Health, Università Cattolica del Sacro Cuore, Rome, Italy; 2 Faculty of Economics, Università Cattolica del Sacro Cuore, Rome, Italy; 3 Steinbeis Europa Zentrum (SEZ), Stuttgart, Germany; 4 Department of Woman and Child Health and Public Health-Public Health Area, Fondazione Policlinico Universitario A. Gemelli IRCCS, Rome, Italy

**Keywords:** Big data, personalized medicine, digital health, European Union, China, policy

## Abstract

**Introduction:**

Several countries are either planning or implementing national strategies for the development and integration of Personalized Medicine (PM) into their healthcare systems. Personalized Medicine is an undisputed priority of the European Commission (EC), which has funded the project “Integrating China into the International Consortium for Personalized Medicine” (IC2PerMed), in order to ensure a common basis for Sino-European collaborations. By mapping the current PM landscape in the European Union (EU) and in China, IC2PerMed aims to provide key solutions toward a synergistic and coordinated approach in the field of PM.

**Methods:**

An extensive desk research was conducted, aimed at identifying documents on PM-related policies, programs, and action plans in the EU and in China, published up to November 2020. The search was conducted by exploring scientific and gray literature, and official institutional repositories. A descriptive summary condensed the information retrieved for both.

**Results:**

Since 2013, the year of publication of the first PM policy by the EC “Use of omics technologies in PM development,” several documents have been published. PM is a key element of the policy agenda also in China, which in 2016 integrated PM into the 13th National Five-Year Plan, followed by the publication of several policies on technology infrastructure and big data. Both in the EU and China, especially in recent years, these policies addressed in detail the issues of big data, data interoperability and exchange, while defining the standards of information and communication infrastructures.

**Conclusions:**

In order to allow optimal collaboration, it is essential to understand similarities and differences between the respective policy strategies, with particular attention to data management and adopted infrastructures. The results of this project may enable the development of joint Sino-European research and innovation initiatives, promoting developments in the field of PM.

## Introduction

Since the completion of the Human Genome Project in 2003, the technological revolution in research and clinical practice has led to a significant transformation of healthcare systems. Such advances have been strongly connected to collecting massive amounts of individual data, including risk factors, lifestyle, genetics, environment, behavior, disease outcomes, and treatment responses. These large and complex datasets, collectively referred to as “big data,” are difficult to process using traditional Information and Communication Technology (ICT) applications and require complex multivariate statistical analysis—“big data analysis/analytics.”

Big data represent a substantial change in the way data are collected and analyzed because they benefit from the technological progress that made possible the computation of such large and complex amounts of data. ICT solutions (such as eHealth, telemedicine, Electronic Health Records [EHRs], Artificial Intelligence [AI]), together with big data analytics tools, are transforming biomedical research and Personalized Medicine (PM). Specifically, PM refers to a medical model using the characterization of individuals’ phenotypes and genotypes (e.g., derived from molecular profiling, medical imaging and lifestyle data) for the purpose of tailoring a therapeutic strategy. The integration of this large quantity of data being produced continuously is essential for a personalized approach to patient needs, as it allows healthcare providers to have a thorough understanding of the patient's condition from their health data, thus using the best available evidence for treating their condition. PM requires advanced technologies and processes to collect, manage, analyze, and interpret the information retrieved, in order to provide targeted support to the right person at the right time. To this end, it is important that this issue is addressed in national and supranational guidelines, policies, and strategies for big data in PM to be effectively used to generate the required knowledge and infrastructure to support new approaches.

Turning PM into an opportunity for all citizens and patients requires the engagement of international stakeholders to define joint research and development approaches, standards, and priorities. This is a crucial priority of the European Commission (EC) research agenda, with funding programs and support actions being developed within the International Consortium for Personalized Medicine (ICPerMed). In this context, the IC2PerMed project (“Integrating China in the International Consortium for Personalized Medicine”) aims to provide critical solutions for enabling the convergence toward a common approach to PM research, innovation, development, and implementation between the EU and China.

The present work, developed within IC2PerMed, aims to identify and summarize the current landscape of PM policies regarding ICT and big data, concerning data management, cross-border data transfer, and data protection in the EU and China.

## Methods

The methods of this work are reported in detail in the Deliverable D1.1 of IC2PerMed entitled “Scoping paper: Review on health research and innovation priorities in Europe and China,” available on the IC2PerMed website. We identified PM policies, strategies, or programs through a three-step desk research.

In the first step, we performed a literature search in PubMed in September 2020 to retrieve articles published in English, with no other restrictions applied, using the keywords “policy, strategy, program, personalized medicine, Europe, China.” Articles that reported information regarding any national laws or legislation in the field of PM at an EU or Chinese level were considered eligible.

In the second step, four authors (IH, TS, MDM, LW) conducted an extensive search in gray literature from October to December 2020, using Google Scholar, Google, and Microsoft Academic search engines, and applying a broad set of search terms, including “policy, strategy, program, personalized medicine, Europe, China” in English, which were then translated into German, Spanish, French, Italian, and Portuguese.

In the third step, four authors (IH, TS, MDM, and SZ) screened national and international official repositories for eligible legislative documents or reports, such as those of the EU Commission and Council, IC2PerMed, and the Ministry of Science and Technology of the People's Republic of China (MoST) and other Chinese public health-related institutions. In addition, relevant documents issued after September 2020 were included in the final version of the manuscript.

A list with the identified documents was created in Excel and subsequently, the following data were extracted: policy name, publication year, country, language, topic, and link. In this manuscript, we focused on PM policies concerning big data and ICT. We conducted a content analysis of the retrieved documents and a descriptive summary, grouping the results into two categories according to whether the documents were issued by EU or Chinese institutions.

## Results

### PM policies issued by EU institutions

The mapping process identified 26 policies issued by EU Institutions dealing with issues related to big data and ICT in the PM field (Table 1). In Europe, policy measures are heterogeneous, being either legally binding or nonbinding. Legally, nonbinding policy measures refer to opinions and recommendations by the EC or conclusions and resolutions by the Council of the European Union aimed at conveying critical strategic positions. Legally binding policy measures include directives (which set compulsory goals for the EU Member States to be implemented in national law) and regulations (legislative acts applied across the EU).

**Table 1.** Descriptive characteristics of eligible EU policies, programs, and action plans.

Policy measure	Year	Main focus
Charter of fundamental rights of the European Union	1950	States the right to protection of personal data as a fundamental right.
Convention 108	1981	Data processing, transborder data flow, quality of data.
Additional Protocol to Convention 108 (ETS No.181)	2001	Additional regulations on transborder data flow and supervisory authorities.
Commission Communication on “eHealth—making healthcare better for European citizens: An action plan for a European eHealth Area” COM (2004) 356 final.	2004	eHealth tools or solutions beyond simply Internet-based applications, including tools for both health authorities and professionals as well as personalized health systems for patients and citizens.
Council conclusions on common values and principles in European Union Health Systems 2006/C 146/01	2006	States the right of all EU citizens to confidentiality of personal information.
Commission recommendation of 2 July 2008 on cross-border interoperability of electronic health record systems 2008/594/EC	2008	Recommendations on the political, organizational, technical, semantic level of cross-border interoperability of EHRs; personal data protection and EHR certification.
Directive 2011/24/EU on the application of patients’ rights in cross-border healthcare	2011	Rules for facilitating the access to safe and high-quality cross-border healthcare. Promotion of collaboration on healthcare between Member States. Responsibilities of Member States regarding cross-border health care.
SWD(2013) 436 Use of “-omics” technologies in the development of personalized medicine	2013	First official document on PM, providing an overview of the state of the art.
REGULATION (EU) No 526/2013 OF THE EUROPEAN PARLIAMENT AND OF THE COUNCIL of 21 May 2013 concerning the European Union Agency for Network and Information Security (ENISA) and repealing Regulation (EC) No 460/2004.	2013	Establishes the EU Agency for Cybersecurity.
Horizon 2020 Work Program, Section “Health, demographic change and wellbeing”	2014	Highlights the relevance of patient and citizen education about ICT applications.
Council conclusions on personalized medicine for patients (2015/C 421/03)	2015	Frames the potential of big data in developing a personalized medicine framework.
Section 8 on “Health, demographic change and wellbeing,” HORIZON 2020—Work Program (2014–2015)	2015	Seven areas on the translational and integrated approach to the challenge of personalizing health and care.
General Data Protection Regulation	2016	EU regulation on data processing and transfer.
Communication on the Mid-Term Review on the implementation of the Digital Single Market Strategy “A Connected Digital Single Market for All” SWD (2017) 155 final	2017	Importance of data being continuously accessible and able to move freely within the single market, accompanied by the necessary high-performance computing capability to analyze it.
Communication from the Commission to the European Parliament, the Council, the European Economic and Social Committee and the Committee of the Regions: European Interoperability Framework—Implementation Strategy COM/2017/0134 final	2017	The European Interoperability Framework (EIF) serves as a common base for EHRs and other digital health applications.
Council conclusions on Health in the Digital Society—making progress in data-driven innovation in the field of health 2017/C 440/05	2017	Citizen empowerment on the use of data they generate.
Section 8 on “Health, demographic change and well-being,” HORIZON 2020—Work Program (2016–2017)	2017	Updates on Section 8 on “Health, demographic change and wellbeing,” HORIZON 2020.
Communication from the Commission to the European Parliament, the Council, the European Economic and Social Committee and the Committee of the Regions on enabling the digital transformation of health and care in the Digital Single Market; empowering citizens and building a healthier society COM/2018/233 final (2018)	2018	Citizens’ secure access to and sharing of health data. Better data to promote research, disease prevention and personalized health and care.
Coordinated Plan on Artificial Intelligence	2018	Common ground for international collaboration on AI development and dedicated investments.
Commission Recommendation (EU) 2019/243 of 6 February 2019 on a European Electronic Health Record exchange format	2019	Principles for access to and cross-border exchange of EHRs.
Regulation (EU) 2019/881 of the European Parliament and of the Council of 17 April 2019 on ENISA and on information and communications technology cybersecurity certification	2019	Reinforcement of ENISA and promotion of the European Cybersecurity Certification.
Council conclusions on shaping Europe's digital future 2020/C 202 I/01	2020	Stresses that individuals, employees and companies in Europe should retain control over their data, based on secure data infrastructures and resilient, trusted value chains, while preserving the EU principle of openness vis à vis third-party countries. Notes that in addition to this, in order to advance personalized and preventive medicine, considerable efforts are required to enable the exchange of health data for research purposes.
Section 8 on “Health, demographic change and wellbeing,” HORIZON 2020—Work Program (2018–2020)	2020	Updates on Section 8 on “Health, demographic change and wellbeing,” HORIZON 2020.
Communication from the Commission to the European Parliament, the Council, the European Economic and Social Committee and the Committee of the Regions—A European strategy for data’ (COM(2020) 66 final) (2020/C 429/38)	2020	Defines the creation of a European data space.
EC Coordinated plan on artificial intelligence Review	2021	Update of the 2018 plan, defining actions and funding instruments for the uptake and development of AI across sectors, including PM
Communication on Fostering a European approach to AI	2021	Further addresses the importance of regulating AI

In the EU, the right to data protection derives from the right to privacy in the *Universal Declaration of Human Rights* (article 12)^1^ incorporated as an individual fundamental right (article 8) in the *Charter of Fundamental Rights of the European Union*,^2^ which specifies that everyone should have the right to data protection and be guaranteed access to their data. The *Treaty on the Functioning of the European Union*—Article 168^3^ highlights the EU's role in encouraging collaboration between the EU Member States in all health-related fields, supporting their actions where necessary, to improve the complementarity of health services in cross-border areas.

The first legally binding international instrument in data protection is *Convention 108*,^4^ which was opened for signature in 1981. Its updated version, *Convention 108 + *^5^ serves as a backbone to EU Member State regulation on data processing, quality, security, and transborder flows, both in the public and private sectors. In 2001, given the increase in personal data exchanges across national borders, parties signed an *Additional Protocol (ETS No.181)*^6^ regarding supervisory authorities and transborder data flows, exercising their functions independently.

*Council conclusions on common values and principles in EU Health Systems 2006/C 146/01*^7^ stated that all EU citizens have the right to privacy and confidentiality of personal information, both in EU and national legislations. Addressing the lack of technical and semantic cross-border interoperability of EHRs has been a priority of the EC, which in the 2008 *Commission Recommendation on cross-border interoperability of electronic health record systems*^8^ advocated that Member States commit to EHR implementation and engage in active collaboration, whereas in the 2019 *Commission Recommendation on a European Electronic Health Record exchange format*^9^ set a baseline for cross-border exchange and interoperability of EHRs while ensuring secure access.

The *Directive 2011/24/EU*^10^ provides rules for facilitating access to safe and high-quality cross-border healthcare and promotes collaboration between the EU Member States. The importance of eHealth was first recognized by the *2004 Commission Communication e-Health-making healthcare better for European citizens: An action plan for a European e-Health Area*,^11^ while in 2016, the *refined eHealth European Interoperability Framework*^12^ was adopted. This framework presents a common approach for managing cross-border and cross-sectoral interoperability and standardization challenges in eHealth. The related eHealth Digital Service Infrastructure ensures the continuity of care for European citizens when traveling abroad in the EU, allowing countries to exchange health data in a secure, efficient, and interoperable way.

In 2013, the EC published the first policy document that recognized the role of ICT in personalized healthcare *“Use of ‘–omics’ technologies in the development of personalized medicine.”*^13^ Subsequently, the need for increased citizen and patient literacy on ICT applications for PM was addressed in the *Horizon 2020 Work Program (2014–2015)*^14^ and then updated in the following *programs of 2016–2017*^15^ and *2018–2020*.^16^ Considering the potential of Artificial Intelligence (AI) to support and enhance the accessibility of ICTs, the 2018 EC *Coordinated plan on artificial intelligence*^17^ underlines the importance of AI systems in clinical decisions and treatment choices, improvement of health image analyses, laboratory or histological data, diagnostic accuracy, and access to healthcare. This plan was then reviewed and updated in 2021 to address better-emerging issues, where health and PM are high-impact sectors that the EU should build strategic leadership in.^18^ The *EC Communication on Fostering a European approach to Artificial Intelligence* further addresses the importance of regulating AI, with relevant implications for the healthcare field, with a proportionate and risk-based European regulatory approach that should cover the different opportunities as well as risks that can stem from the future implementation of AI in healthcare.^19^

In the *Council conclusions on Personalized Medicine for patients (2015/C 421/03)*,^20^ the EU Council invited the EC to investigate, under the Third Health Program (2014–2020), how to realize the potential of big data in PM, while also considering ethical, legal, and social aspects. The rise in big data requires optimal cybersecurity practices, and in this regard, the EU Agency for Network Information Security (ENISA), established by *Regulation (EU) No 526/2013 of the European Parliament and Council*,^21^ has the most relevant tasks in matters of cybersecurity. The 2019 *Regulation on ENISA*^22^ clarifies its duties, promoting the use of the European cybersecurity certification and increasing security and transparency.

The EC is currently working on a European health data space, a system for data exchange and access under ordinary rules, procedures, and technical standards to ensure that health data can be accessed within and between the Member States, with full respect to the fundamental rights of individuals and in line with the *General Data Protection Regulation (GDPR)*.^23^ The GDPR represents the most advanced legislation on EU data regulation, processing, and free movement to date (Art. 1). It entered into force in 2016 and became applicable across all Member States on May 25, 2018, repealing the preexisting Directives 95/46/EC and 02/58/EC. The GDPR principles regarding health data are further detailed within the *Council conclusions on Health in the Digital Society 2017/C 440/05*.^24^

The *EC Communications on Digital Single Market*^25^ recognize the importance of making health data generated in the EU freely accessible and further encouraging the improvement of data quality and access to pursue personalized health care. As stated in the *Council conclusions on shaping Europe's digital future*,^26^ data ownership and governance should be guaranteed while preserving openness with third-party countries and making efforts to enable the exchange of health data for research purposes. The *European strategy for data*^27^ explicates the aim of creating a single market for data that will ensure Europe's global competitiveness and data sovereignty.

### PM policies issued by Chinese institutions

Our mapping identified 16 policies issued by Chinese Institutions dealing with issues related to big data and ICT in the PM field (Table 2). In China, laws, regulations, strategic outlines, guiding opinions, and normative standards convey policy measures from different organizations issuing the documents. The administrative system in China is vertical and managed by the central government, which distributes indications to regions, provinces, municipalities, and other local administrative centers. Consequently, all the leading players in China in the field of PM are related to the government, among which the MoST and the State Council play a key role. The Chinese ecosystem is deeply connected to its government, which bases national growth prospects on five-year plans. These plans include the objectives on which policies, action plans, programs, and laws in China will be developed and defined. The most recent five-year plan includes new projects aiming to improve the population's health level.

**Table 2.** Descriptive characteristics of eligible Chinese policies, programs, and action plans.

Policy measure	Year	Main focus
12th Five-Year plan	2011	Promotes the development of health services.
Management Measures on Population Health Information	2014	Management of security and privacy protection of citizen health data.
Outline of Action to Promote the Development of Big Data	2015	Promotes information and containment systems for public data to develop health services.
Notice of the Council of State on the issuance of the action plan	2015	Defines big data and the regulation for collection and use in the medical field.
13th Five-Year Plan	2016	Introduces the role of Big Data platforms in Precision Medicine and promotes the application of emerging technologies, such as genetic testing.
Healthy China 2030	2016	Introduces Precision Medicine into citizen health services and strengthening of key technological breakthroughs.
Guiding Opinions on Promoting and Regulating the Development of Big Data Applications for Health Care	2016	Integration, sharing, and application of healthcare and medical big data.
National Innovation-driven Development Strategy Outline	2016	Support for innovative and technological innovations aimed at Precision Medicine and genetic screening technologies.
Guiding Opinions on Promoting and Regulating the Development of Health and Medical Big Data Applications	2016	Medical and health data are considered a fundamental national resource. Data use and sharing are considered a national priority.
Notice of the State Council on the issuance of the action plan for the promotion of the development of Big Data	2016	Promotes development and application of big data in China with the creation of a central data collection system.
Cyber Security Law of the People's Republic of China	2017	Regulation on cyberspace safety and matters of national security.
National Health and Medical Big Data Standards, Safety and Service Management Measures / Health Committee	2018	Purpose, basis, scope of application of big data, introducing a management method.
Opinions on Promoting the Development of “Internet + Medical Health”	2018	Guidelines for the adoption of innovative medical service models.
National New Generation Artificial Intelligence Standard System Construction Guide	2020	Strengthens the design of high-level standardization in the field of artificial intelligence.
Guidelines for the construction of a new generation standard artificial intelligence system	2020	Promotes a standard system of new generation artificial intelligence, healthy, and sustainable.
Personal Information Protection Law of the People's Republic of China	2021	China's regulation on data processing and transfer.

The *12th Five-Year Plan*^28^ significantly improved basic medical insurance while calling for better hospital and clinical infrastructure, including management, public health and medical education, and greater use of information technologies in healthcare. This five-year plan also attaches equal importance to Chinese traditional (typically regarded as the most widespread kind of medicine) and Western medicine.

In 2014, the National Health Commission published the *Management Measures on Population Health Information [Trial]*,^29^ which standardizes the management of health and medical information to improve accountability and power consistent with ensuring safety, convenience, and efficiency. Medical and health services and family planning institutions at all levels are responsible for collecting, using, managing, securing, and protecting the privacy of the population's health information.

The State Council's 2016 *Guiding Opinions on Promoting and Regulating the Development of Big Data Applications for Health Care*^30^ present a model for sharing health and medical data on a national platform, which contains medical and health information to achieve interdepartmental and interregional sharing of primary data resources. This guide takes up and deepens the *Notice of the State Council on Issuing the Action Plan for Promoting the Development of Big Data*,^31^ which promotes the development of big data, explains the importance of data collection and use, and organizes their modality. The *2018 National Health and Medical Big Data Standards, Safety and Service Management Measures*,^32^ delivered by the Health Committee, define the purpose, basis, and scope of application of big data, introducing a management strategy.

The *Outline of Action to Promote the Development of Big Data*^33^ by the State Council promotes information and containment systems for public data interconnected with government data to support the development and application of big data.

The *Personal Information Protection Law of the People's Republic of China (PIPL)*^34^ is China's primary privacy law, providing a comprehensive framework regulating data privacy within the Chinese territory and outside of it. In 2016, the State Council issued the *Guiding Opinions on Promoting and Regulating the Development of Health and Medical Big Data Applications*,^30^ which defines medical and health data as a fundamental national resource. It coordinates and standardizes the legislative directives at the governmental and local level, accelerating the creation of a data market that is oriented to the international commercial environment. In 2017, the Standing Committee of the National People's Congress issued the *Cyber Security Law of the People's Republic of China*,^35^ which ensures cybersecurity, guaranteeing cyberspace sovereignty and national security. The *13th 5-Year Plan*^36^ introduces the “Technological Innovation for Health and Healthcare,” which is focused on implementing the strategy of innovation-driven growth, boosting technological innovations and their application, including mobile internet, cloud computing, and big data, and ensuring balanced progress. High-level standardization in AI, research, and development in the field was promoted by two guidelines, published in 2015^37^ and 2017,^38^ and reinforced in the *Standardization Administration of the Central Information Office Network National Development and Reform Commission, Ministry of Industry and Information Technology on the issuance of “a new generation of artificial intelligence, national notification system construction guidance standard”*^39^ published in 2020.

The Outline of the *Healthy China 2030 Plan*,^40^ issued in 2016, aims to build a health and medical big data application system, promote the open sharing and wide use of such data through platforms of regional population health information, and coordinate the deployment of national biomedical big data. The 2018 *Opinions on Promoting the Development of “Internet + Medical Health”*^41^ by the State Council highlight the importance of strengthening data protection activities and warn that sensitive data, such as patient information, need to be stored in China. The *National Health and Medical Big Data Standards, Safety and Service Management Measures / Health Committee [Trial]*^32^ encourages the development of technologies to protect public data and provide resources for increasing citizens’ access to medical services and imposes sanctions should these not be respected.

The *National Innovation-driven Development Strategy Outline*^42^ puts forward the goal of including China among the innovative countries, expanding its economic strength by focusing on technological progress, science, and talents as a strategic resource in policy formulation and institutional agreements.

## Discussion

The field of PM is progressing at an astonishing pace, riding the wave of exponential progress in technological improvements and scientific research. Nonetheless, policymakers are keeping up with these advancements, both in the EU and China, issuing a broad spectrum of policies and programs to guide and address PM in the best way possible.

Our mapping identified several policies that recognize the importance of new technologies for better health, paying attention to how users and stakeholders apply them and addressing relevant issues such as data safety, governance, and related digital infrastructure assessment.

At the EU level, the EC and the European Council have issued many directives, communications, conclusions, treaties, and regulations. In contrast, in China, many institutional entities, including the State Council and the MoST, have produced policies, initiatives, and other directives, which are then approved by the central government, followed by national or local dissemination. This decentralized EU approach is accountable for much of the variation among EU Member States, given the national level differences in laws, regulations, policy planning, and implementation, each following or interpreting the EU norms in their own way, looking at ways to embrace the new possibilities emerging in the field of PM.

The EU and China share a common focus on several critical issues in PM, including the centrality of the person in the care pathway; the effectiveness of tailored prevention, diagnosis, and treatment; the collection and use of health data; and the harmonization of technical standards to ensure interoperability and cross-border exchanges. On both sides, this convergence can be seen as a necessity in renewing the healthcare systems, together steering toward a personalized approach with tailored treatment strategies for individual patient subgroups, with a stronger focus on disease prevention as a response to increasing healthcare costs.

Personalization of the medical approach dates to the early 2000s, with the introduction of personalized health system concepts for patients and citizens in the EU in 2004^43^ and the discussion around precision surgery in China in 2006.^44^ In 2015–2016, personalized or precision medicine was defined, respectively, in the EU^20^ and China,^45^ leading to the development of programs for implementing—omics technologies and precision/personalized prevention and treatment. A unified definition of PM is not available yet; therefore, these concepts are currently used interchangeably.

The role of big data in the field of PM was considered in 2016, both by the EU^24^ and China,^33^ with policies and guiding opinions issued on the regulation of the application of health and medical big data. Considering that the revolution of PM is related to the capability of storing, merging, and accessing large quantities of data without jeopardizing the owners’ safety and privacy, several policies in the last few years have been dedicated to these aspects. In the EU, their focus was set on ensuring data FAIRness (Findability, Accessibility, Interoperability, and Reusability),^9^ providing a European Interoperability Framework^12^ for EHRs and digital health applications within the EU and among MS, and creating a single data market^25^ and a common European Data Space.^27^ In China, the Guiding Opinions on Promoting and Regulating the Development of Big Data Applications for Health Care policy of 2016^35,46^ set clear rules for data governance, mentioning the creation of a national platform to collect, organize and store health data at national, provincial, municipal, and county level while integrating it with other data sources and removing any barriers. The Cyber Security Law^35^ is the central policy on data protection and states that personal information and important data gathered or produced within China shall be stored within China and may only leave the country following a security assessment by the State cybersecurity and informatization departments.

Data transfer within the EU is free as long as it meets the conditions stated under the GDPR. As for data transfer outside the EU, the same conditions as within the EU need to be fulfilled. The EC may adopt an adequacy decision regarding a third-party country, stating that data may flow from the EU to that third-party country without any further safeguards being necessary, assimilating it *de facto.* As of now, neither China has received an adequacy decision nor has the EC launched a procedure for adopting an adequacy decision for transfers of personal data to China.^47^ Therefore, any data transfer from the EU to China would need precautions. The situation is similar when it comes to data transfer within China and from China to a foreign country, including the EU Member States: such data transfer is regulated by the PIPL, with stringent requirements on security controls and data localization, especially when such data are transferred abroad.

China and Europe are at the forefront of this “PM rush,” with increasing numbers of projects, publications, and patents produced yearly in this area. Within the global landscape, the EU is generally well-placed in the application of AI in the healthcare domain, somewhat behind China but on a par with the USA.^48^ The EU is characterized by a strong research dimension, with research institutions accounting for about two-thirds of all EU players in this field. In comparison, academic and research institutions only make up one-third of players in China and a relatively small proportion in the US, where commercial companies are dominating developments in this field.^48^

PM is very promising in the matter of improved health outcomes and healthcare systems’ sustainability, but it requires more intense global collaboration for different entities to come together and join their efforts. From our work, we highlighted how many policies and programs are directed at increasing collaboration opportunities and trying to align different stakeholders’ priorities. Nonetheless, the road ahead and the many opportunities laid out in this field are such that facilitations allowing collaboration between the EU and China are needed. Key elements that should be addressed thoroughly by policymakers include interoperability between different organizations; eHealth solutions; collaborative work between systems or entities; and harmonization and standardization of cross-border data sharing and transfer processes. Ensuring analyzable, comparable, and merged data across borders is a complex issue requiring stakeholders at different levels to agree on common standards. Defining common goals will also be a key aspect in laying the groundwork for future collaboration: whereas drugs and medical devices are designed upon concrete indications, dealing with big data and its requisite infrastructure requires the creation of specific indications. A Sino-European agreement would help to overcome the restrictions imposed on transfers to non-European countries by the GDPR in Europe and a series of restrictive regulations in China, including the recently introduced PIPL.^47^ If this was the case, it is easy to imagine the benefits that would result from the amount and variety of data made available to researchers, clinicians, and healthcare workers alike, with benefits stemming both on the professional and the patient side.

In the US, there is still a fragmented policy framework concerning big data, reflecting that legislation can hardly keep pace with the rapid developments in the field.^49,50^ The lack of regulations, incentives, and systems to manage ownership and responsibilities hinders its clinical application.

Our results should be considered in light of some limitations. Despite the extensive search in institutional sources, and in scientific and gray literature, both European and Chinese, we do not presume to have retrieved all the possible documents on the topic, especially the ones not publicly available, including draft proposals or ones yet to be published at the time of our research. Although this could suggest the presence of a publication bias, to our knowledge, our work is the first attempt to identify PM-related policies on big data management, ICT, cross-border data transfer protection, and legislation in the EU and China. By providing an overview of policies and strategies, we might help inform stakeholders about future Sino-European collaboration and synergies.

## Conclusion

To conclude, our mapping showed that both the EU and China recognize the importance of PM implementation, share common interests, and aim at reaching shared goals. Despite this, our research highlighted a lack of synergy between their paths, which could lead to wasted effort. Besides the differences in regulatory frameworks, future approaches will need to consider cultural and social differences, not to mention ethical issues.

To shift the future of medicine toward a personalized approach, increased collaboration between relevant stakeholders is necessary, creating more Sino-European cross-border policies and partnerships. Outlining the regulatory profiles might help provide new perspectives and directions to PM-related initiatives and programs; our comparative analysis might, therefore, help to provide the basis for further collaboration between the EU and China, strengthening their leadership in PM on a global level.
